# A Magnetoencephalographic/Encephalographic (MEG/EEG) Brain-Computer Interface Driver for Interactive iOS Mobile Videogame Applications Utilizing the Hadoop Ecosystem, MongoDB, and Cassandra NoSQL Databases

**DOI:** 10.3390/diseases6040089

**Published:** 2018-09-28

**Authors:** Wilbert McClay

**Affiliations:** 1 Information Systems Department, Northeastern University, Boston, MA 02115, USA; mcclay.w@husky.neu.edu; Tel.: +1-571-272-8012; 2 Department School of Social Work, Tulane University School of Medicine, New Orleans, LA 70112, USA; 3 Department of Information Assurance, Northeastern University, Boston, MA 02115, USA; 4 Lawrence Livermore National Laboratory, Livermore, CA 94550, USA; 5 Department of Information Assurance, Brandeis University, Waltham, MA 02453, USA; 6 Department of Mathematics, Brandeis University, Waltham, MA 02453, USA

**Keywords:** brain-computer interface, machine learning algorithms, encephalographic (EEG), magnetoencephalographic (MEG), Hadoop Ecosystem, iOS Mobile Applications, MongoDB, Cassandra, Emotiv EPOC headset, OpenVibe

## Abstract

In Phase I, we collected data on five subjects yielding over 90% positive performance in Magnetoencephalographic (MEG) mid-and post-movement activity. In addition, a driver was developed that substituted the actions of the Brain Computer Interface (BCI) as mouse button presses for real-time use in visual simulations. The process was interfaced to a flight visualization demonstration utilizing left or right brainwave thought movement, the user experiences, the aircraft turning in the chosen direction, or on iOS Mobile Warfighter Videogame application. The BCI’s data analytics of a subject’s MEG brain waves and flight visualization performance videogame analytics were stored and analyzed using the Hadoop Ecosystem as a quick retrieval data warehouse. In Phase II portion of the project involves the Emotiv Encephalographic (EEG) Wireless Brain–Computer interfaces (BCIs) allow for people to establish a novel communication channel between the human brain and a machine, in this case, an iOS Mobile Application(s). The EEG BCI utilizes advanced and novel machine learning algorithms, as well as the Spark Directed Acyclic Graph (DAG), Cassandra NoSQL database environment, and also the competitor NoSQL MongoDB database for housing BCI analytics of subject’s response and users’ intent illustrated for both MEG/EEG brainwave signal acquisition. The wireless EEG signals that were acquired from the OpenVibe and the Emotiv EPOC headset can be connected via Bluetooth to an iPhone utilizing a thin Client architecture. The use of NoSQL databases were chosen because of its schema-less architecture and Map Reduce computational paradigm algorithm for housing a user’s brain signals from each referencing sensor. Thus, in the near future, if multiple users are playing on an online network connection and an MEG/EEG sensor fails, or if the connection is lost from the smartphone and the webserver due to low battery power or failed data transmission, it will not nullify the NoSQL document-oriented (MongoDB) or column-oriented Cassandra databases. Additionally, NoSQL databases have fast querying and indexing methodologies, which are perfect for online game analytics and technology. In Phase II, we collected data on five MEG subjects, yielding over 90% positive performance on iOS Mobile Applications with Objective-C and C++, however on EEG signals utilized on three subjects with the Emotiv wireless headsets and (*n* < 10) subjects from the OpenVibe EEG database the Variational Bayesian Factor Analysis Algorithm (VBFA) yielded below 60% performance and we are currently pursuing extending the VBFA algorithm to work in the time-frequency domain referred to as VBFA-TF to enhance EEG performance in the near future. The novel usage of NoSQL databases, Cassandra and MongoDB, were the primary main enhancements of the BCI Phase II MEG/EEG brain signal data acquisition, queries, and rapid analytics, with MapReduce and Spark DAG demonstrating future implications for next generation biometric MEG/EEG NoSQL databases.

## 1. Introduction

In Phase I, “*A Real-Time Magnetoencephalography Brain-Computer Interface Using Interactive 3D-Visualization and the Hadoop Ecosystem*”, Journal of Brain Sciences, 2015 [1], was developed with an overall above 92% performance on 5 MEG subjects utilizing the Variational Bayesian Factor Analysis (VBFA) Machine Learning algorithm.

**Figure 1:**
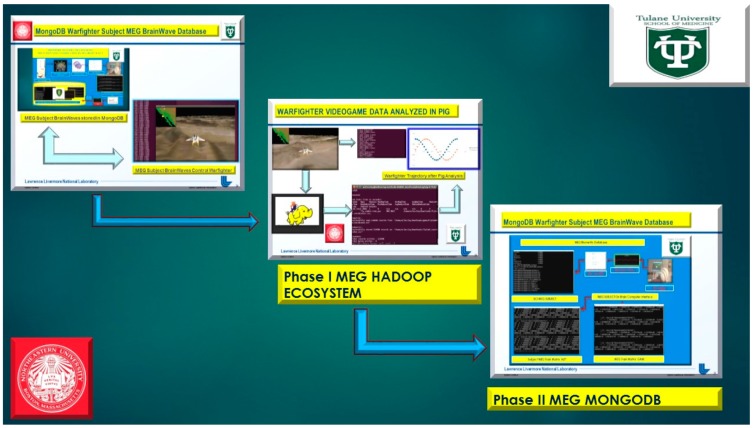
Phase II, MongoDB MEG Brain Computer Interface Database(s).

**Figure 2:**
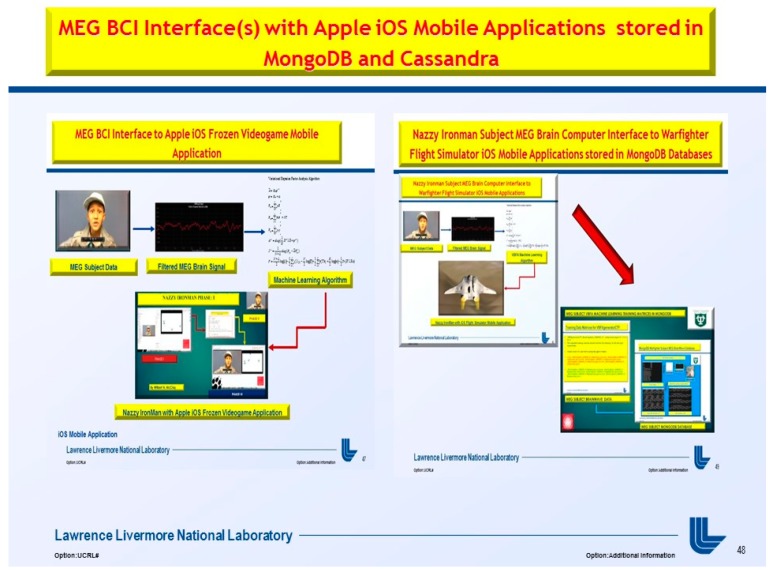
Phase II, magnetoencephalography brain-computer interface(s) (MEG BCI) with Apple iOS Mobile Applications stored in MongoDB and Cassandra.

Secondly in Phase II, demonstrate in Figure 1 and Figure 2, below, we extend the MEG Subjects’ brainwave [2] data storage to MongoDB for ease of use and relevance with the Internet of Things (IoTs) sensor based data acquisition [3] for analytical processing and data storage in Figure 2, extended to implementation with Apple iOS Mobile Applications.

Thus, the acquisitioned tomographic magnetoencephalography/electroencephalography (MEG/EEG) subject brainwave signals can be uploaded locally on a single node or into a new and innovative cloud based hosting infrastructure referenced as a Cloud Service Provider (CSP) utilizing MongoDB and/or Cassandra as the NoSQL databases.

The previous tomographic MEG/EEG subject brainwave data analysis locally stored acquisitioned brainwave signals into directories in a UNIX based file system or utilizing a relational database system, such as PostgreSQL or MySQL. However, this proved unfruitful for real-time data acquisition for subject’s brain signals and was focused on a RAID (Redundant Array of Disks) architecture. The main fallbacks and hazards with utilizing a Relational Database Management System for acquired subject’s brain signals in real-time is if a MEG/EEG electrode array or sensor fails to acquisition signal data from the subject it will result in nulls in the Relational Database Management System (RDBMS). Furthermore, if the Database System Administrator attempts to run queries with a RDBMS filled with null values it will cause major castrophes and possible crash the entire system, particularly for interactive queries on large datasets.

The innovative and next generation usage of NoSQL databases, referenced as MongoDB and Cassandra, where the rows and columns in the database do not need to be uniform as in a RDBMS, and is also schema-less architecture, where the rows and columns do not require uniformity.

For instance, this provides a distinct advantage for real-time brain signal acquisition, because, if a sensor fails, the MongoDB NoSQL database is not compromised, and additionally MongoDB uses “Replication and Sharding” for distributed computing where the data is separated into blocks or chunks and spread across distributed machines. Likewise, CASSANDRA NoSQL database is a column-oriented NoSQL database designed by Facebook and distinctly designed for fast reads and writes, fault-tolerance, and elasticity, and is more favorable than most NoSQL databases in that domain. In terms of certain aspects of performance, the CASSANDRA NoSQL database is column-oriented NoSQL database and has faster reads, writes, and elasticity than MongoDB, but MongoDB is more popular and versatile for the usage of Internet of Things (IoTs) devices (e.g. sensors, MEG/EEG brain signal electrode array sensors) [2,3].

The concept of extracting brainwaves and classification of user intent is referred to as brain-computer interfaces (BCIs), sometimes called mind-machine interfacing (MMI) or brain-machine interfacing (BMI), has been evolving for many years. These interfaces are used for both noninvasive procedures (such as magnetoencephalography (MEG) and electroencephalography (EEG)), as well as for invasive procedures (such as electrocorticographic (ECoG) events). What follows is a brief discussion of the history and importance of BCIs in the noninvasive procedures of MEG and EEG as they relate to recent applications, ranging from interactive video game technology to robotics and mobile applications.

One of the most dynamic current applications of these BCI developments is, “User Input Validation and Verification for Augmented and Mixed Reality Experiences”, created by Aylin Climenser, Hani Awni, Frank Chester Irving, Jr., and Stefanie A. Hutka, under United States Patent Publication Number: US2018/0188807A1 [4]. The premise utilizes a head mounted display (HMD) incorporating an EEG to monitor and analyze event related potentials (ERP) to a given stimulus that was observed by the subject. Additionally demonstrated in Illustration C, Yongwook Chae with LOOXID LABS, INC., was issued patent US2018/0196511, “EYE-BRAIN INTERFACE (ERI) SYSTEM AND METHOD FOR CONTROLLING SAME”, [5], on 12 July 2018, where the system is defined as providing an eye-brain calibration (EBC) interface for calibrating eye movements and brain waves simultaneously, in addition, the EBC interface is composed of a visual object and instructs a user to focus or gauze into the visual object in a particular cognitive state; thus acquisitioning eye movements and brainwaves of the user for the visual object that was included in the EBC.

**Figure 3:**
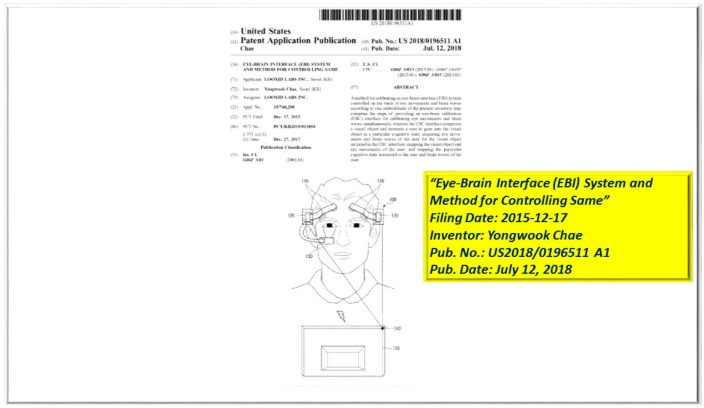
Yongwook Chae, “EYE-BRAIN INTERFACE (ERI) SYSTEM AND METHOD FOR CONTROLLING SAME”, US2018/0196511.

Furthermore, Cruz-Hernandez at Immersion Corporation in 2018 was issued patent US 20170199569 A1, entitled, “Systems and methods for haptically-enabled neural interfaces”, the processor is configured to receive a sensor signal from a neural interface configured to detect an electrical signal that is associated with a nervous system demonstrated in Figure 3, above. Additionally, the processor is utilized to detect an interaction with a virtual object in a virtual environment based on the sensor signal [4].

As a result of the innovation of the next generation MEG and EEG BCI applications, this technology is potentially applicable to other types of cloud based architectures utilizing Big Data analytics to reduce computational time and expense. The general consensus is that such methods could yield the same result with less time lag and more compact BMI devices [2].

**Figure 4:**
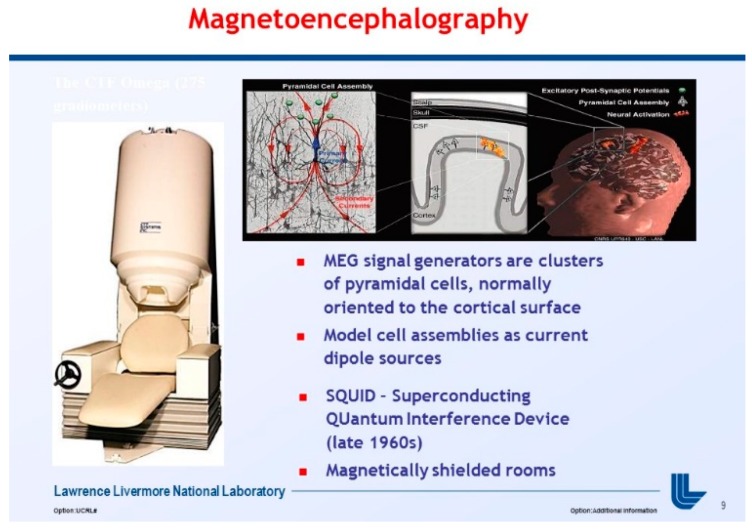
University of San Francisco in California (UCSF) MEG Scanner with Superconducting Quantum Interference Device (SQUID) detectors.

**Figure 5:**
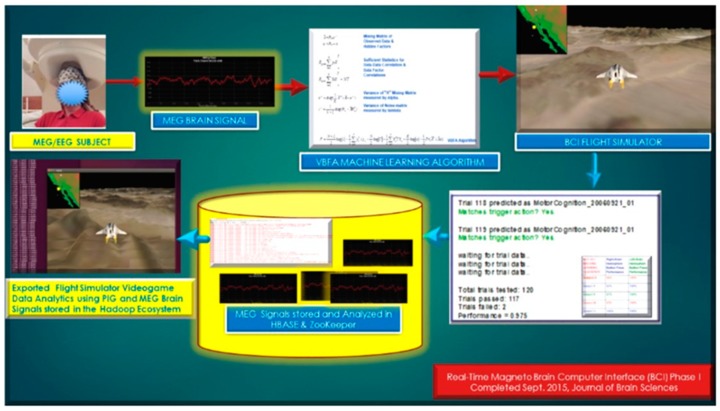
Phase I, “A Real-Time Magnetoencephalography Brain-Computer Interface Using Interactive three-dimensional 3D-Visualization and the Hadoop Ecosystem”, Journal of Brain Sciences, 2015.

**Figure 6:**
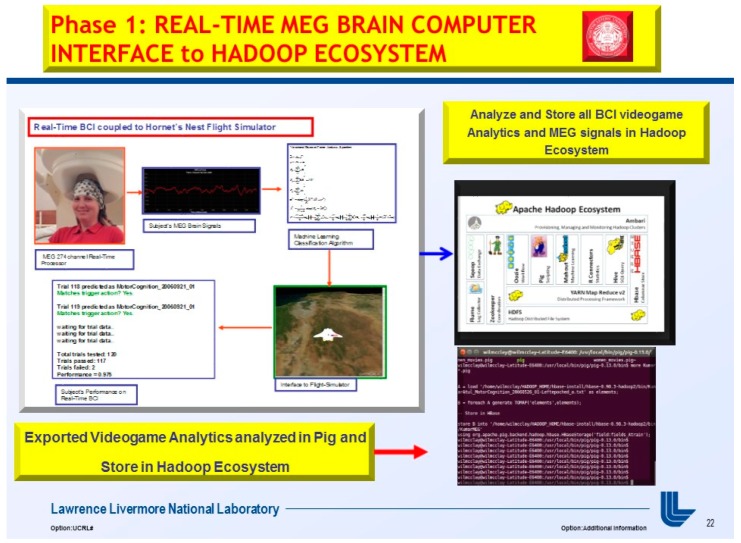
Phase I, “A Real-Time Magnetoencephalography Brain-Computer Interface Using Interactive 3D-Visualization and the Hadoop Ecosystem”, flowchart process of BCI analytics in the Hadoop Ecosystem.

At the moment, the integration of the gaming industry, mobile, and Big Data analytics was approximated at $137.9 billion for 2018 [6]. The use of NoSql databases, such as Cassandra, MongoDB, and the Hadoop Ecosystem yields keen competitive advantages over legacy relational transactional databases, and web-based games are now the go-to standard platform with the brisk adoption of mobile games [7]. Thus, an MEG based Brain-computer Interface (BCI) utilizing videogame analytics attracts two primary audiences: (1) the neuroscience and neuro-engineering scientific community and (2) gaming and Big Data analytics industry. The market revenue for BCI applications interfaced to videogames has unparalleled future market revenue for avid gamers and diligent research scientists. Furthermore, the videogame analytics and processing were stored in the Hadoop Ecosystem demonstrated in Figure 1 and Figure 2 (above), Figure 4, and below in Figure 5 and Figure 6.

Currently, the telemedicine & healthcare industry has now adopted gamification, or the utilization of game mechanics and design, to inspire people for motivation and behavioral influence that is focused on wellness and healthy behaviors [7].

## 2. UCSF MEG System

At the University of California, San Francisco (UCSF), MEG technology is being used to study multimodal and multiscale imaging of dynamic brain function as well as cortical spatiotemporal plasticity in humans [1,8]. Thus, several novel signal processing and machine learning algorithms had to be constructed, and UCSF had to fully utilize its twin 37-channel bio magnetometer. This machine uses 275 channel SQUIDS-based detectors, housed in a magnetically shielded room (MSR), to noninvasively detect tiny magnetic fields generated by neuronal activity in the brain, as demonstrated in Figure 4, below.

From these signals, computational modeling allows a spatiotemporal view of the time course and spatial patterns of neuronal activity. The UCSF lab also uses digital 64-channel EEG and three-dimensional (3D) computing facilities [1].

**Figure 7:**
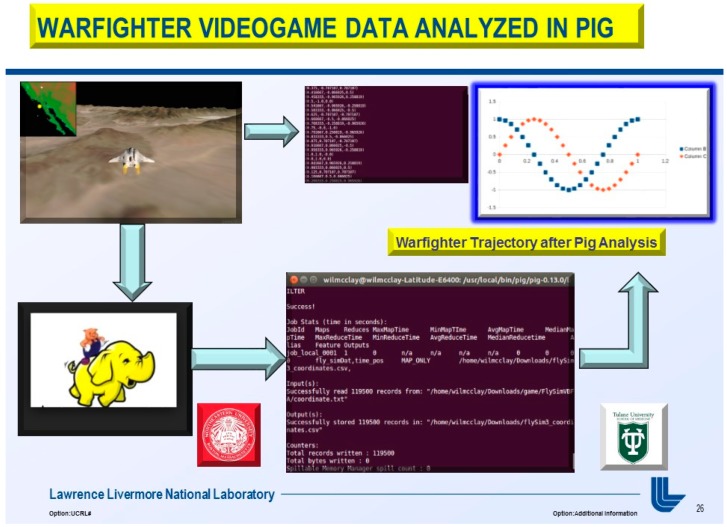
Phase I, “A Real-Time Magnetoencephalography Brain-Computer Interface Using Interactive 3D-Visualization and the Hadoop Ecosystem”, Pig analysis for MEG Subject performance on Warfighter.

**Figure 8:**
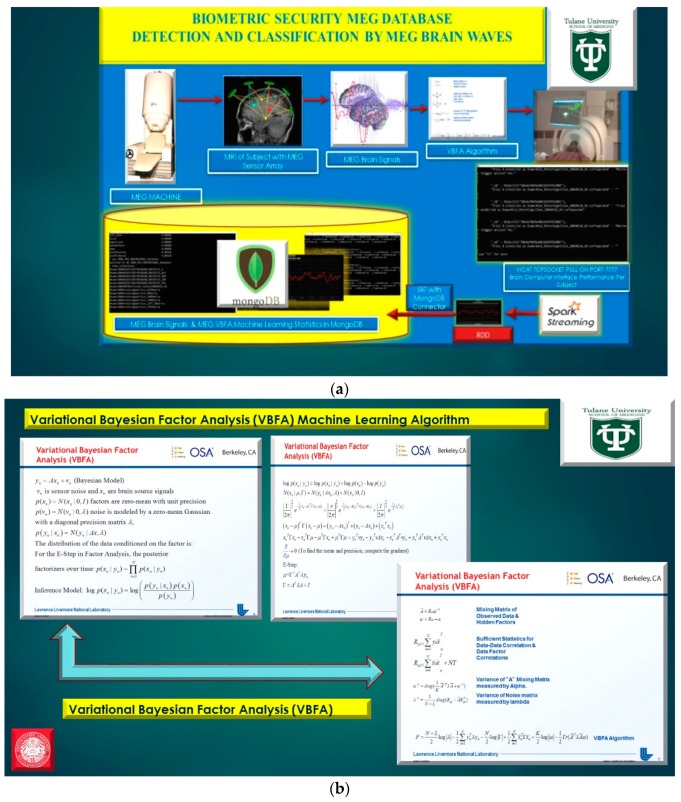

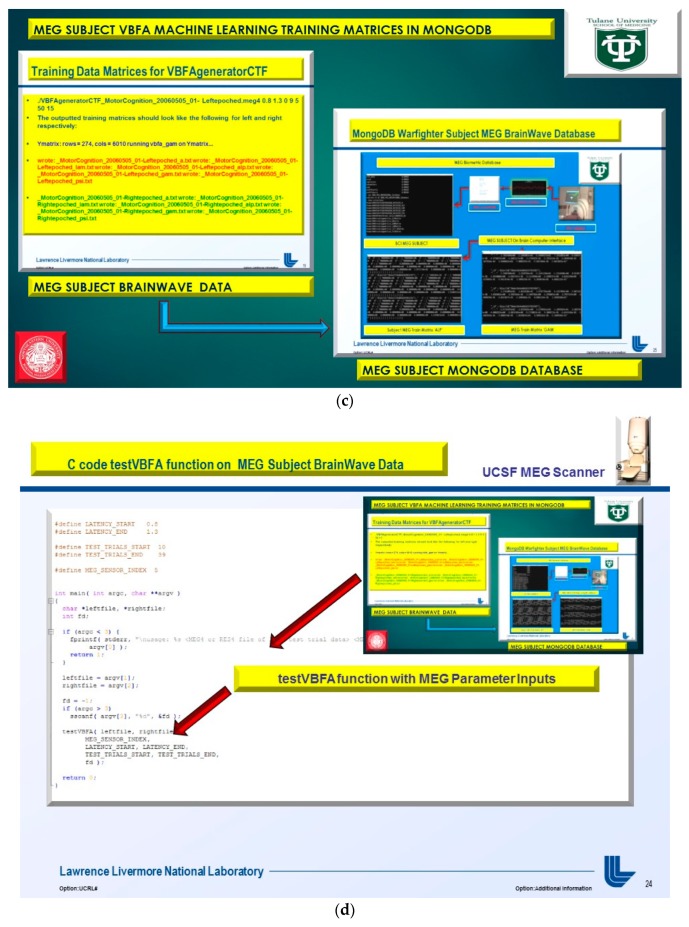
(**a**) Phase II, MongoDB Magnetoencephalography Brain-Computer Interface Database. (**b**) Phase II, Variational Bayesian Factor Analysis (VBFA) Machine Learning Algorithm. (**c**) Phase II, MEG Subject Brain Wave Data and VBFAgeneratorCTF training matrices in MongoDBdatabase(s). (**d**) Phase II, C code testVBFA function on MEG Subject Brainwave Data.

The manuscript utilizes the Variational Bayesian Factor Analysis (VBFA) machine learning algorithm to classify and detect MEG/EEG brainwave classification [9,10] to define the user’s intent while viewing a flight-simulator videogame in real-time [1], demonstrated in Figure 7 and Figure 8. Furthermore, prestigious neuroscientists, such as Srikantan Nagarajan at University of San Francisco in California (UCSF), utilized location bias and spatial resolution in the reconstruction of a single dipole source utilizing various spatial filtering techniques in neuromagnetic imaging. The analysis of location bias for myriads of representative adaptive and non-adaptive spatial filters that are based on their resolution kernels validating standardized low-resolution electromagnetic tomography referenced as (sLORETA) using a minimum-variance spatial filter for MEG source reconstruction [9,10,11,12,13,14].

## 3. Phase II: Wireless EEG MongoDB & Cassandra Brain Computer Interface Databases and iOS Applications

**Figure 9:**
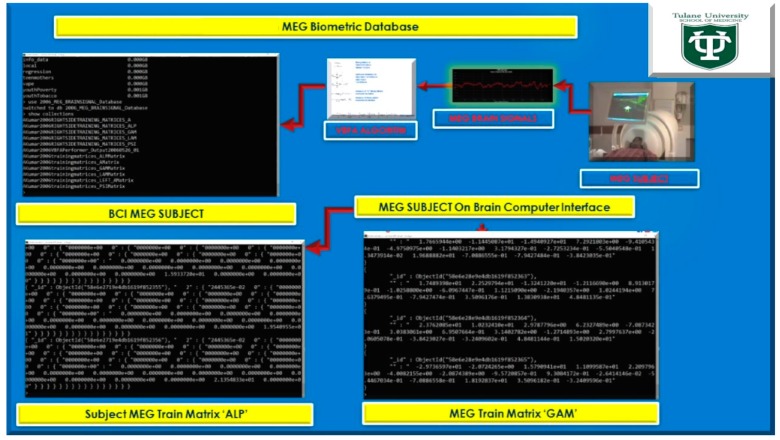
Phase II, MongoDB Magnetoencephalography Brain-Computer Interface Database storage of MEG Subject Variational Bayesian Factor Analysis training matrices and MEG Subject Performance and Metadata.

**Figure 10:**
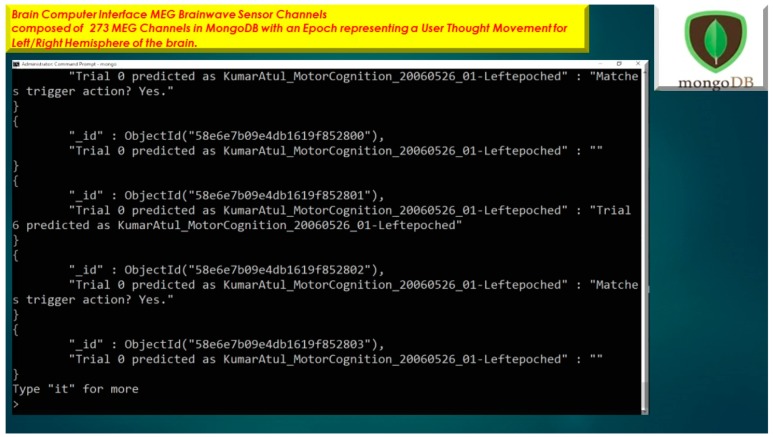
MEG Brainwave data acquisition in MongoDB with 12-byte BSON timestamp representing ObjectID for Epoch Trial performance for MEG Subject.

**Figure 11:**
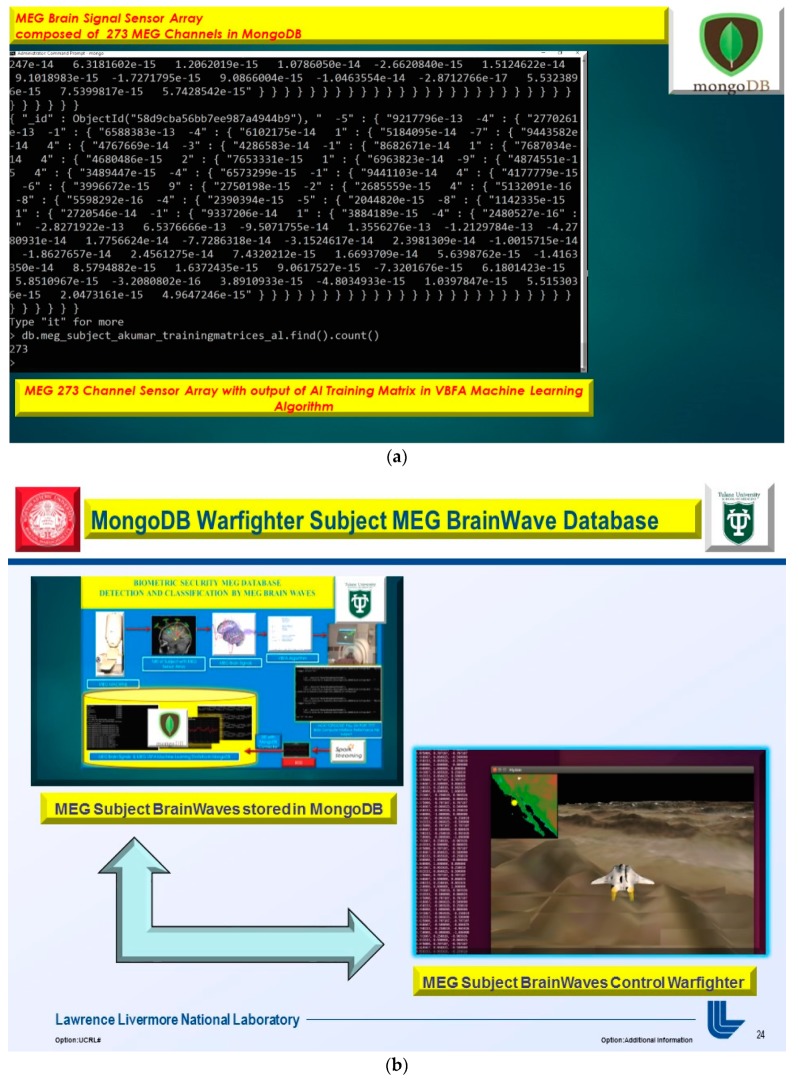

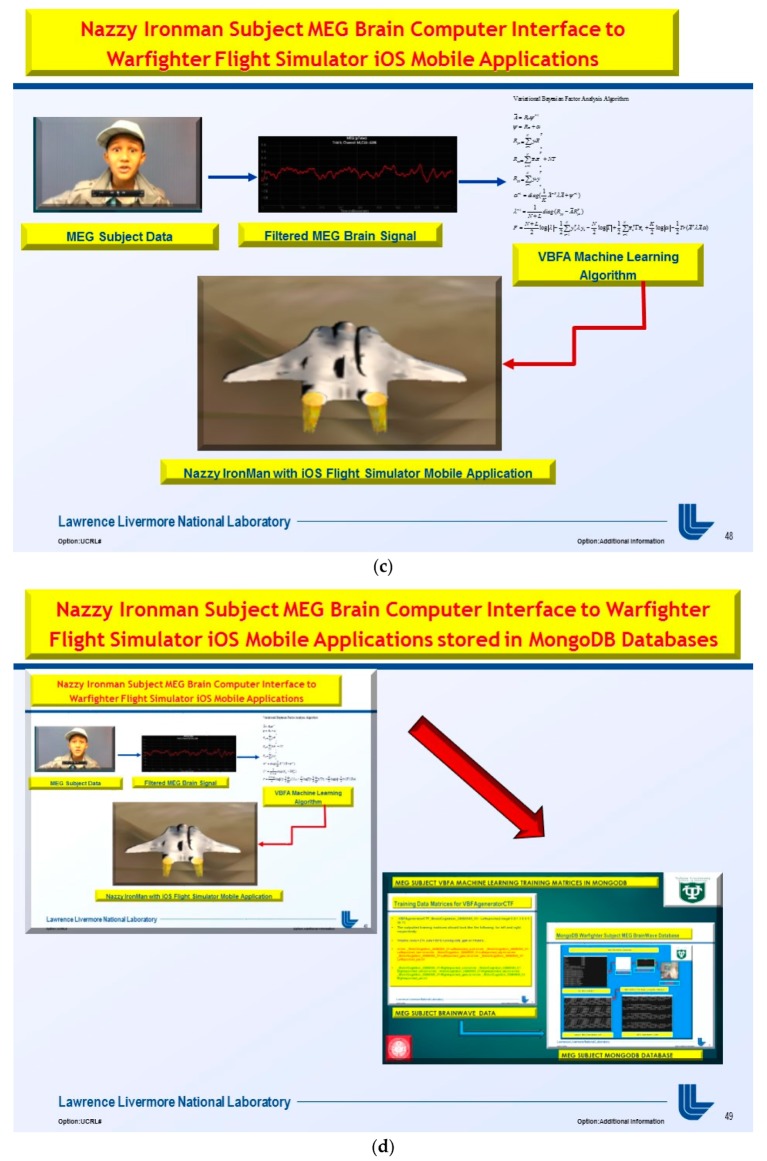
(**a**) MEG Brainwave data acquisition in MongoDB with 12-byte BSON timestamp representing ObjectID representing Subject’s Training Matrices acquired during VBFA Machine learning algorithm training on MEG brainwaves. (**b**) MEG Brainwave data acquisition in MongoDB with 12-byte BSON timestamp representing ObjectID representing with Subject Brainwaves controlling flight of Warfighter simulation. (**c**) Nazzy Ironman Subject MEG Brain Computer Interface to Warfighter Flight Simulator iOS Mobile Applications yielding over 90% performance on MEG Subject brain signal data. (**d**) Nazzy Ironman Subject MEG Brain Computer Interface to Warfighter Flight Simulator iOS Mobile Applications stored in MongoDB databases yielding over 90% performance on Subject Data, demonstrated in Figure 9, Figure 10 and Figure 11.

**Figure 12:**
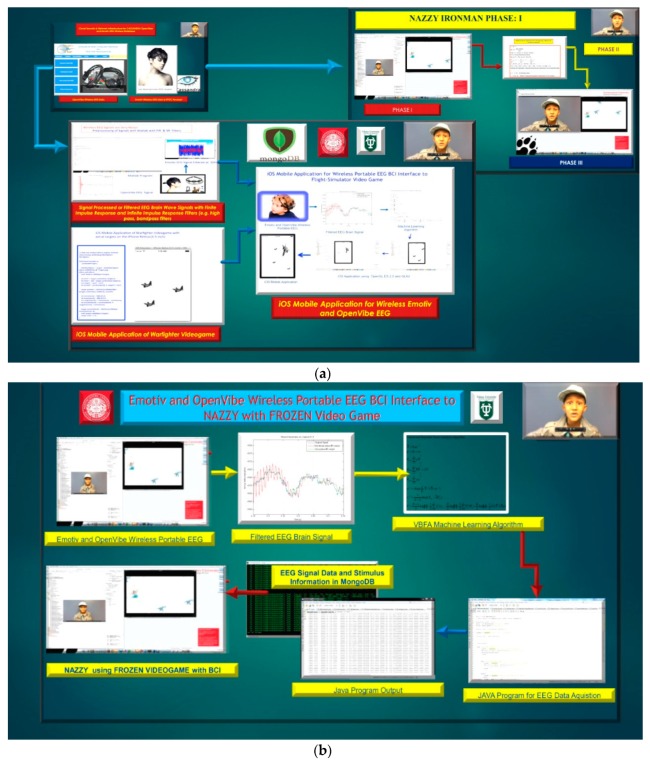
(**a**) NAZZY IronMan with Frozen Videogame & iOS Warfighter Mobile Game for Brain Computer Interface Project with Emotiv/OpenVibe Wireless electroencephalography (EEG) brain signal(s) data while using machine learning algorithms to classify brain signals in iOS videogame applications utilizing EEG brain signal data storage in NoSQL database MongoDB. (**b**) NAZZY IronMan with Frozen Project with Emotiv Wireless EEG brain signal(s) data using machine learning algorithms to classify brain signals in iOS Frozen videogame utilizing EEG brain signal data storage in NoSQL database MongoDB.

The Phase II integrates with Phase I utilizing the same machine learning classifier as in Phase I project begins with the use of a wireless Emotiv Epoch EEG headset integrated to MongoDB and Cassandra NoSQL databases with iOS Mobile Applications, demonstrated in Figure 9, Figure 10 and Figure 11. Figure 12, illustrates the overall and analogous architecture of the Phase II NAZZY with Frozen Video Game BCI process. The Emotiv Epoch EEG headset was designed by Emotiv Systems the predominant leader in commercial Brain Computer Interface technology. The Emotiv Epoch system was utilized to measure the electrical activity that is associated with the brain and muscles of the face and it converts brain signals and activity into control signals [1,10]. The Emotiv Epoch implements Artificial Neural Networks based learning and training techniques while using the McCulloch-Pitts model and Back-Propagation Neural Network algorithm [10].

### 3.1. EEG Data Acquisition and Signal Processing

The aim of the wireless BCI research assumes that it is possible to assess thought movements of individuals that are induced by specific events in the virtual environment while using EEG and videogame analysis. In it is stated mental thoughts which are characteristics of user intent can be classified using machine learning algorithms, while the subject’s brain waves are being acquired via wireless EEG, and are essential in this research for measuring multiple states from left-right thought movement. Electroencephalography (EEG) is the recording of electrical brain activity, which is caused by the firing of neurons within the brain. The EEG brain signals and machine learning/signal processing feature extraction are used to classify the EEG signal will be used as a control signal for the warfighter simulator or NAZZY Frozen Video game. The classification of EEG brainwave data illustrating multiple states of left-right thought movement are detected and analyzed with novel machine learning algorithms. The output of the subject’s analytics while playing the flight simulator videogame or NAZZY Frozen Videogame post signal classification is recorded and warehoused in a NoSQL Cassandra and MongoDB database environment.

**Figure 13:**
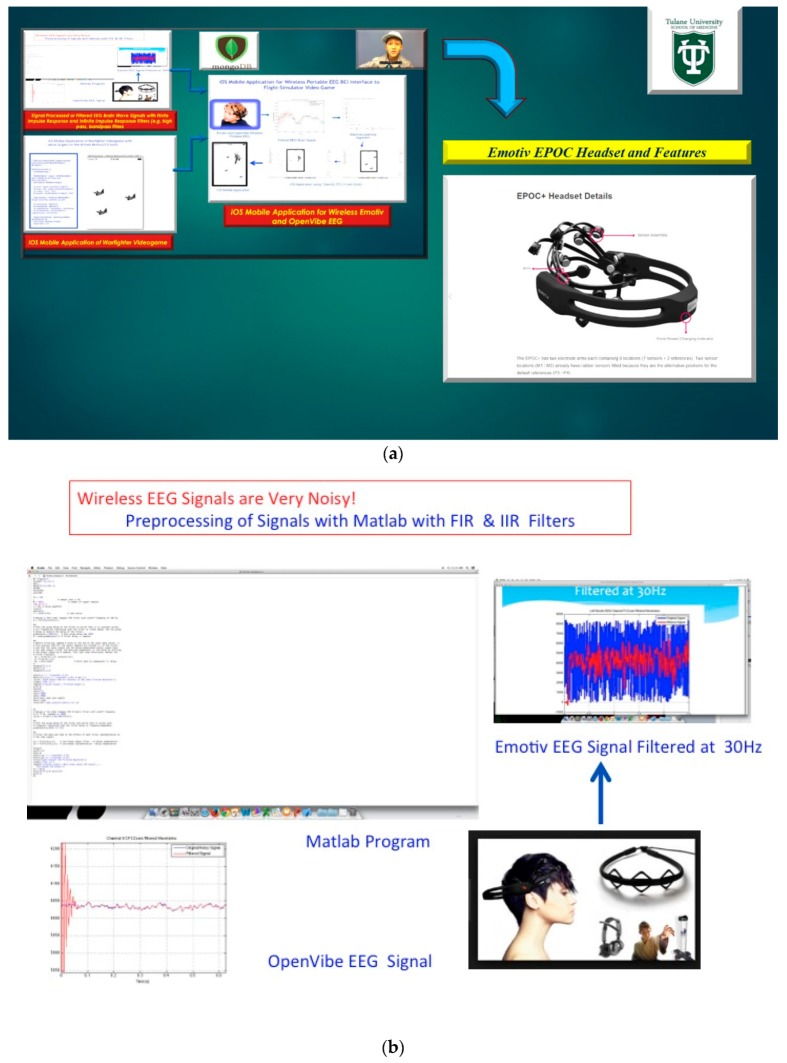
(**a**) Emotiv EPOC Headset, Features, and Brain Computer Interface applications. (**b**) Utilization of Matlab FIR (Finite Impulse Response) & IIR (Infinite Impulse Response) Bandpass and Lowpass Filters on Wireless EEG Signals.

The dilemma with wireless EEG is that the sensors typically may have a very poor signal-to-noise ratio (e.g., lower monetary EEG sensor array cost may yield poor conductivity) and the utilization of proprietary software (e.g., Emotiv Back-Propagation Neural Networks) are often necessary to pre-process the wireless EEG brain-wave signals before the VBFA machine learning classifier can be applied, see in Figure 13 [15]. The lack of conductivity from EEG sensor array as opposed to MEG sensor arrays are based on a higher remuneration cost for design for brain signal acquisition. In the human brain, the current is generated mostly by pumping the positive ions of sodium, potassium, calcium, and the negative ion of chlorine, through the neuron membranes in the direction that is governed by the membrane potential [16]. Thus, an EEG signal is a measurement of currents during synaptic excitations of neurons in the cerebral cortex. The human head pertains to three different layers inclusive of the skull, scalp, and brain, and myriads of subsequent thin layers. The lack of conductivity from EEG sensors to acquisition brain signals is due to the skull attenuating the signals approximately one hundred times more than soft tissue [10,16].

EEG is the quintessential tool to study and diagnosis myriads of neurological disorders and other brain related abnormalities. The utilization of EEG Brain Computer Interface technology has myriads of applications to the following [1,17]:

#### Brain-Machine Interfaces

- Pilots and flight control
- Vigilance monitoring for air force, navy, or ground troop vehicles
- Clinical settings: Monitoring patient mental states and providing feedback
- Education: Improving vigilance, attention, learning, and memory
- Monitoring mental processes (“reading the mind”)
- Detecting deception (FBI, CIA, other law enforcement agencies)
- Predicting behavior
- Detecting brain-based predispositions to certain mental tendencies (the brain version of Myers-Briggs)
- Likelihood of improving with one type of training versus another
- Likelihood of performing better under specific circumstances

### 3.2. EEG Cassandra NoSQL Databases

**Figure 14:**
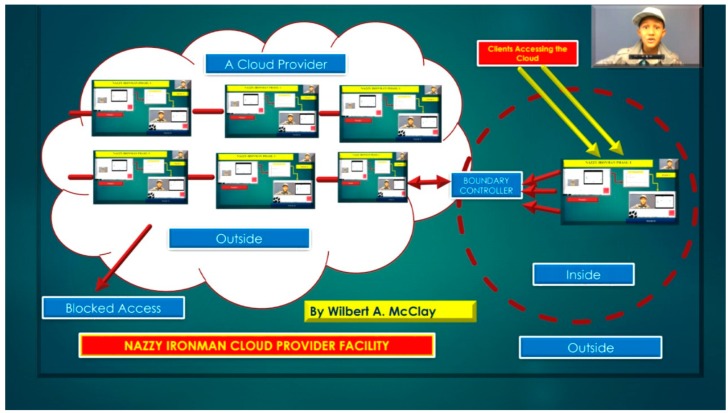
Nazzy IronMan Brain Computer Interface Cloud Provider Facility with Cassandra NoSQL database(s).

**Figure 15:**
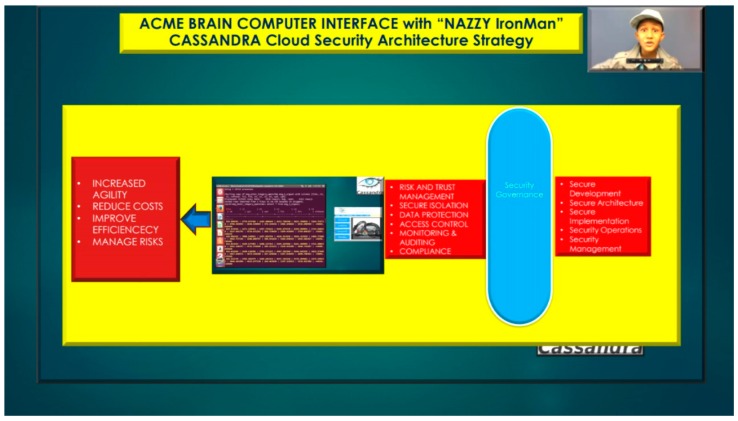
Nazzy IronMan Brain Computer Interface Cassandra Cloud Security Architecture Strategy.

Apache Cassandra is a proven high availability and scalable NoSQL database without diminishing in performance. The NoSQL database, Cassandra, is “suitable for applications that can’t afford to lose data, even when an entire data center goes down [17]”. Therefore, with respect to Brain Computer Interface applications where brainwave signals are acquired in an EEG/MEG electrode array referencing each sensor channel often in real-time. The utilization of the Cassandra NoSQL column-oriented architecture database is a quintessential solution due to fault-tolerance, decentralization indicating no individual failure points and that every node is identical with data automatically replicated [18,19]. The usage of elasticity is a necessity with the Cassandra NoSQL database, since read and write output have linearity as new BCI machines could be added to the cloud network, as illustrated in Figure 14 and Figure 15, below discussing beneficial cloud security constraints.

### 3.3. Cassandra EEG Databases

#### Cassandra EEG Databases: KeySpaces and Column-Families

**Figure 16:**
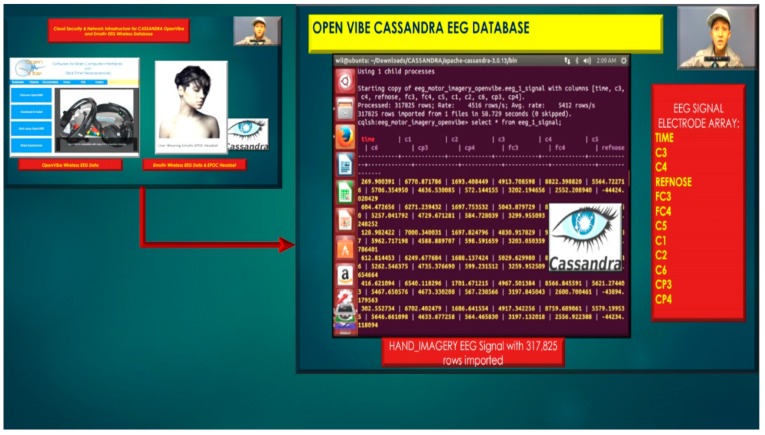
Emotiv and OpenVibe EEG Sensor Array stored in Cassandra NoSQL database.

The Cassandra NoSQL database(s) utilizes KeySpaces (referenced as databases) for the BCI EEG project the referenced KeySpace, eeg_motor_imagery_openvibe illustrated in Figure 16, utilizes a Simple Strategy which is referred to as “Rack Unware Strategy”, the strategy utilizes by default in the org.apache.cassandra.locator.RackUnwareStrategy configuration file. The Simple Strategy places replica sets in a single data center in a topology, which is “not aware” of their placement on a data center rack, thus denoted “Rack Unware Strategy”. Theoretically, the usage of Rack Unware Strategy is computationally faster in theory due to implementation, but this is not the case if the next data node has the given keys as opposed to other data nodes.

**Figure 17:**
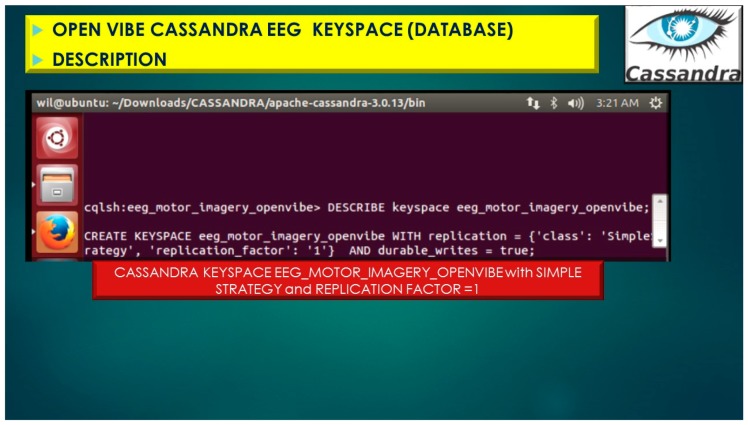
OpenVibe EEG Sensor Array stored in Cassandra NoSQL KEYSPACE (database) with Simple_Strategy and Replication Factor = 1.

**Figure 18:**
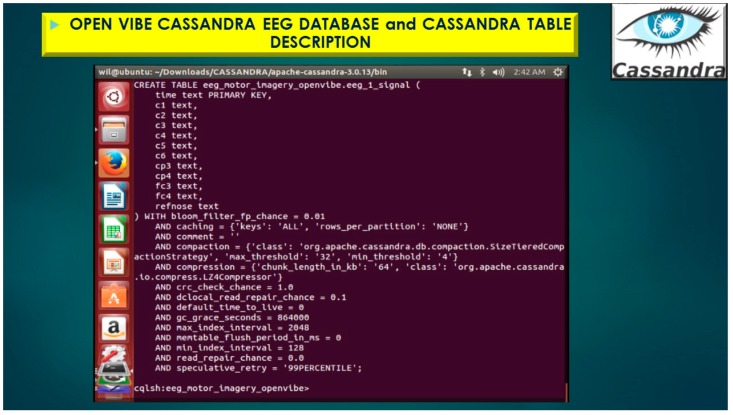
OpenVibe EEG Sensor Array stored in Cassandra NoSQL KEYSPACE (database) with Simple_Strategy and Replication Factor = 1 displaying primary key and all attributes for keyspace, eeg_motor_imagery_openvibe and table, eeg_1_signal Cassandra statistics.

The Replication Factor is specifically important because it indicates how many copies of the portioned data will be stored and then distributed and dispersed throughout the Cassandra Cluster. The Replication Factor Setting yields this information indicated as the following, Simple_Strategy and Replication Factor = 1, and demonstrated in Figure 17 and Figure 18, with displaying primary key and all attributes for keyspace, eeg_motor_imagery_openvibe and column-family (table), eeg_1.

Furthermore, if the replication factor is set to 1, then the writes are written only to a single node, as in Figure 17 and Figure 18, below. If the nodes goes down, the values are no longer accessible. However, if the replication factor is set to 2 or greater, then the nodes in the cluster will get the value written to the nodes on every write and therefore replicas of each other.

**Figure 19:**
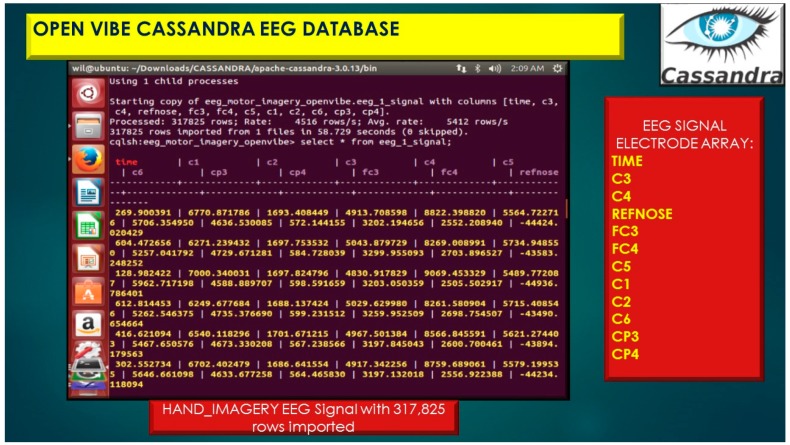
OpenVibe EEG Sensor Array stored in Cassandra NoSQL KEYSPACE (database) with Simple_Strategy, table, eeg_1_signal importing 317,825 rows of EEG brain signal data.

**Figure 20:**
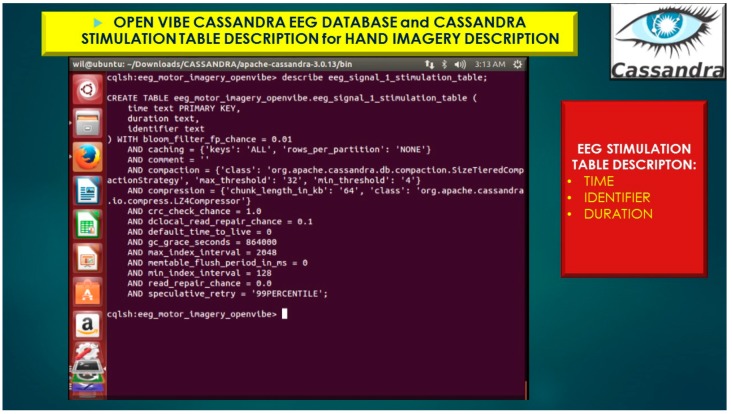
OpenVibe EEG Sensor Array stored in Cassandra NoSQL KEYSPACE (database) with Simple_Strategy, Stimulation table, eeg_signal_1_stimulation_table importing eeg brain signal data (*e.g., time, identifier, duration*).

The eeg_1 represents the Cassandra Column-Family illustrated in Figure 19 and Figure 20, which is analogous to a table in a traditional relational database system.

### 3.4. EEG MongoDB NoSQL Databases

**Figure 21:**
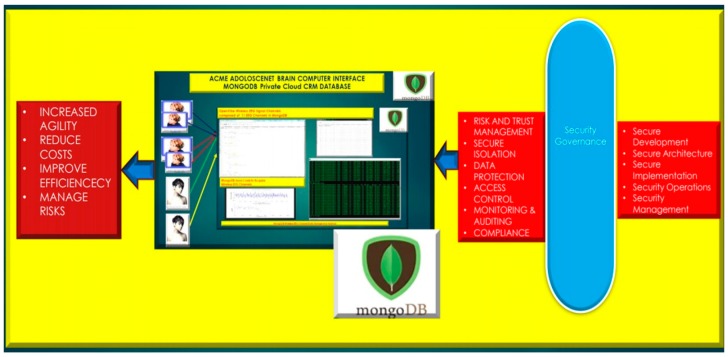
MongoDB Brain Computer Interface Cloud Security Restraints.

**Figure 22:**
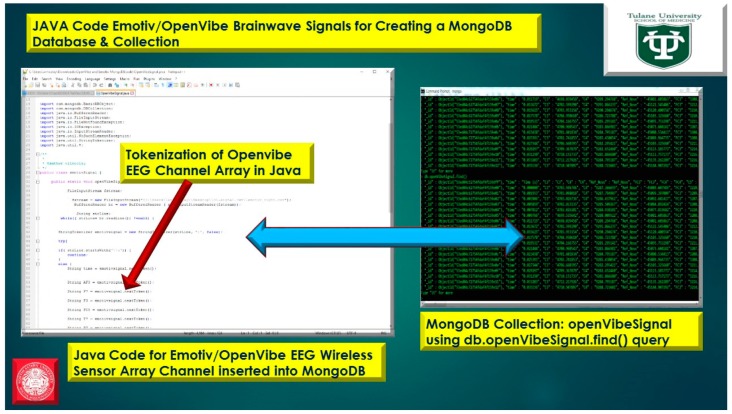
Java Tokenization of OpenVibe EEG Sensor Array inputted into MongoDB Collection utilizing db.openVibeSignal.find() queries.

**Figure 23:**
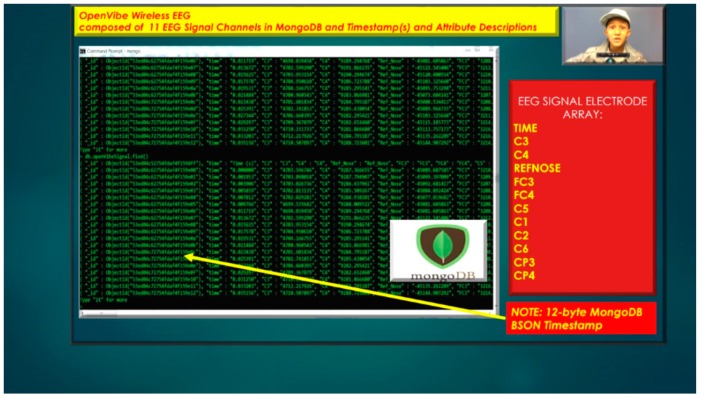
Usage of NoSQL database MongoDB for Wireless EEG Signal Storage and Retrieval with MongoDB BSON Timestamp with EEG Signal Electrode Array.

**Figure 24:**
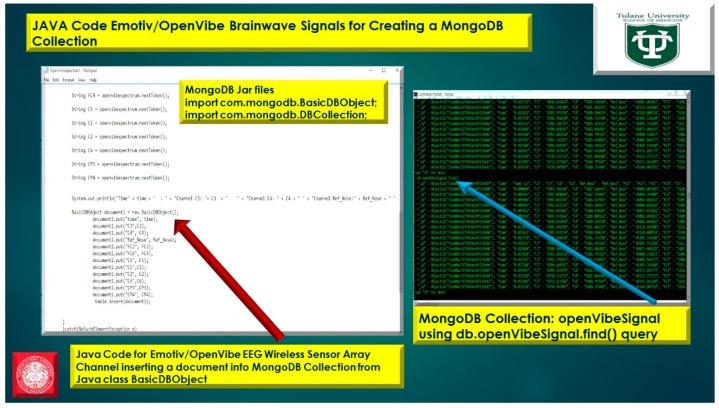
Java Program for Emotiv and OpenVibe EEG Sensor Array Channel inserting a document into MongoDB Collection using Java class ***BasicDBObject***.

**Figure 25:**
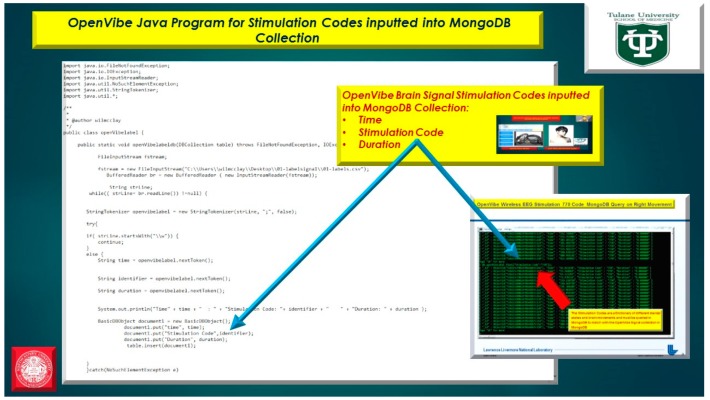
OpenVibe EEG Sensor Array Java Program for Brainwave Signal Stimulation Codes for time, stimulation code, and duration.

**Figure 26:**
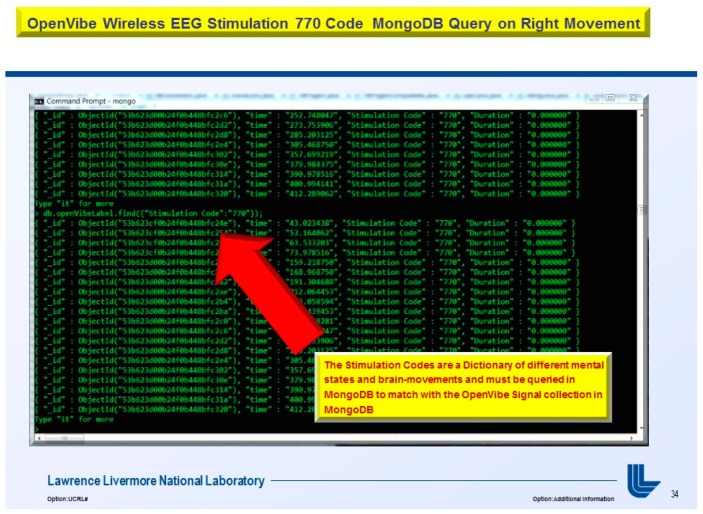
Wireless EEG Java Stimulation Code Dictionary to input EEG signal patterns in MongoDB.

**Figure 27:**
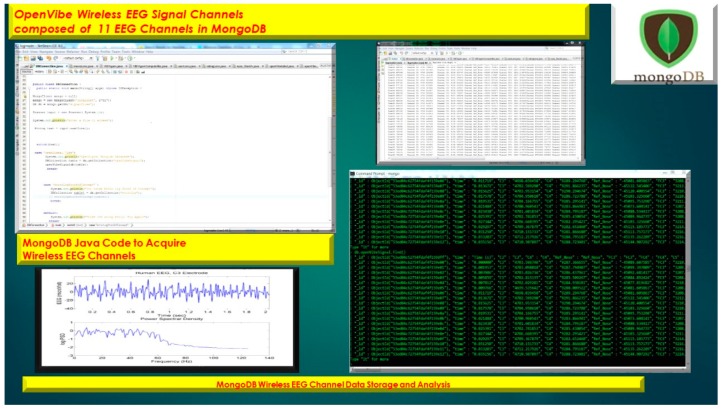
Stimulation Codes have to match the acquired EEG signal patterns in MongoDB.

The usage of a NoSQL database such as MongoDB are excellent for fast queries and indexing without the usage of a schema-less architecture and is perfect for sensor inputs, particularly if the sensor fails are does not acquisition the signal properly it will yield a null value which for large number of users could be devasting to a typical relational database management system. The novelty of the MongoDB fast indexing and querying shown in Figure 21, Figure 22, Figure 23, Figure 24, Figure 25, Figure 26 and Figure 27, below illustrates the power of MongoDB to match the user’s intent or Stimulation Code against the acquired brain-signal for fast indexing. As in subsequent sections, the usage of elasticity is a necessity with the MongoDB NoSQL database, since read and write output have linearity as new BCI machines are added to the cloud network, as illustrated in Figure 21 below, discussing beneficial cloud security constraints.

**Figure 28:**
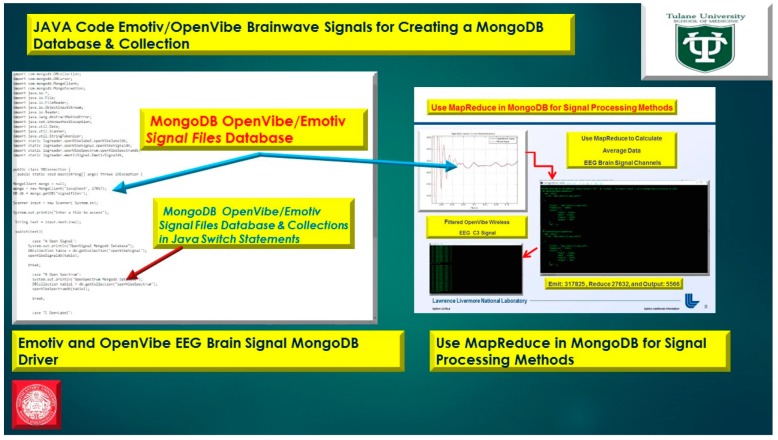
MapReduce in MongoDB for Signal Processing and EEG data analytics.

The other asset to utilizing MongoDB as a document-oriented data store is the use of MapReduce as a computational paradigm for key-value pairs to do basic signal processing methods for each user’s intent in a condensed and formulated manner, as shown below in Figure 23, Figure 24, Figure 25, Figure 26, Figure 27 and Figure 28. MongoDB also utilizes an ObjectID with a 12-byte timestamp in BSON notation composed of the machine id, process, and other features. The usage of ObjectID to identify a document in a MongoDB collection (e.g., table) is useful for MEG/EEG brainwave signal channel arrays, which can be easily parsed in a Java MongoDB driver file to Tokenize the channel array before ingestion into the MongoDB, “Signal Files” database, as demonstrated in Figure 23 [20,21], below. For instance, in Figure 23, a MongoDB connection was implemented with a series of Java Case Switch Statements illustrating either an Emotiv or OpenVibe collection for ingestion into the MongoDB Signal Files database, also illustrated in Figure 27. In addition in Figure 23, once the OpenVibe EEG sensor channel is Tokenized and ingested in MongoDB, the usage of signal processing techniques on EEG channels can be implemented in MongoDB also utilizing the MapReduce computational paradigm algorithm for key-value pair analysis with signal processing techniques illustrated in Figure 28, below. Thus, this is beneficial for sensor-based signal acquisition, analysis, and fault-tolerance. For instance, if one of the MEG or EEG sensor channels fails to emit brain signal activity, the database will not yield large sequences of “NULL” values that could easily compromise or crash a traditional relational database during signal acquisition and real-time queries. Therefore, the utilization of NoSQL databases such as MongoDB are the quintessential tool for real-time signal acquisition and analysis for data storage [2], and additionally with the MongoDB BSON timestamp notation a replica data set on a node can be easily referenced and detected if a system failure took place during the Brain Computer Interface brainwave data acquisition.

### 3.5. EEG and MEG BCI Objective and iPhone Integration

**Figure 29:**
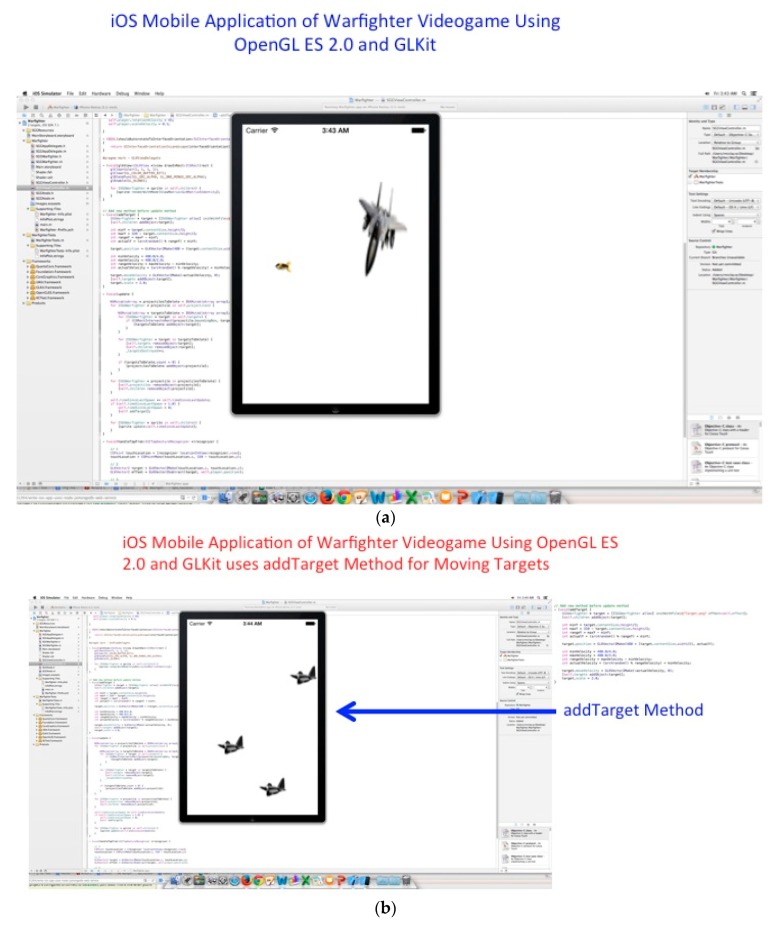

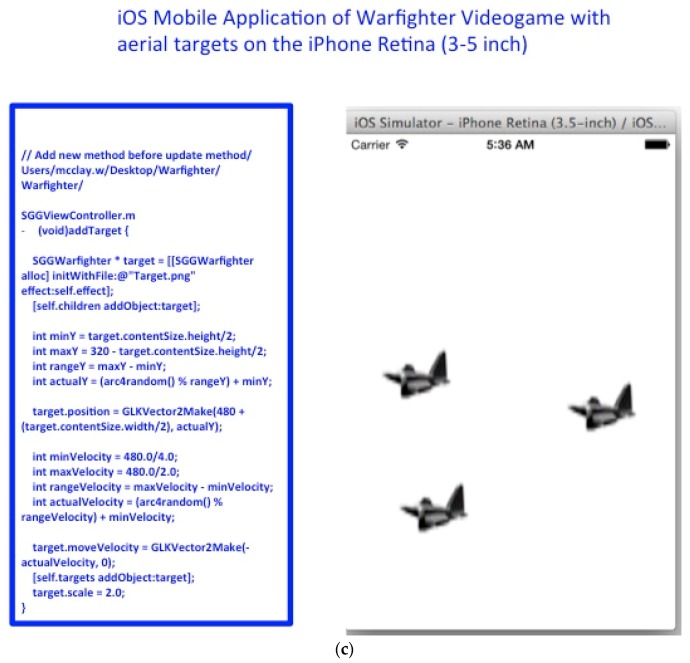
(**a**) iOS Mobile Application of Warfighter Videogame using OpenGL ES 2.0 (Khronos Group, Beaverton, Oregon if USA, country, https://www.khronos.org/about/) and GLKit with the UITapGestureRecognizer class to fire a projectile. (**b**) iOS Mobile Application of Warfighter Videogame using OpenGL ES 2.0 and GLKit with aerial targets using the addTarget Method. (**c**) Display of iOS Mobile Application of Warfighter Videogame using OpenGL ES 2.0 and GLKit with aerial targets using the addTarget Method (close-up).

**Figure 30:**
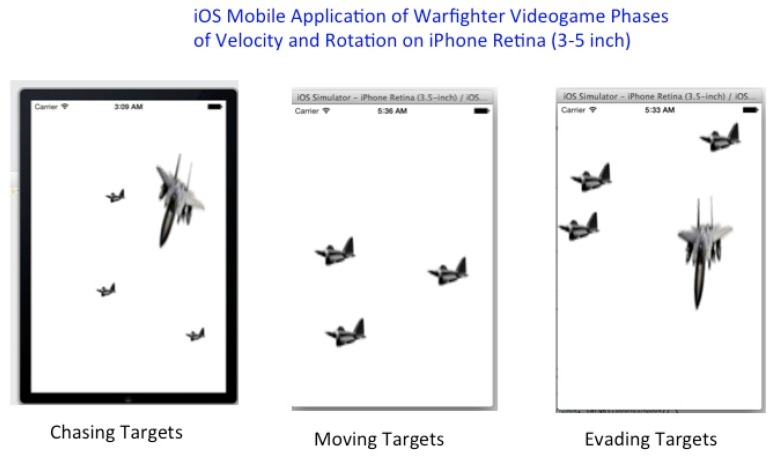
iOS Mobile Application of Warfighter Videogame using OpenGL ES 2.0 and GLKit to evade or chase aerial targets.

**Figure 31:**
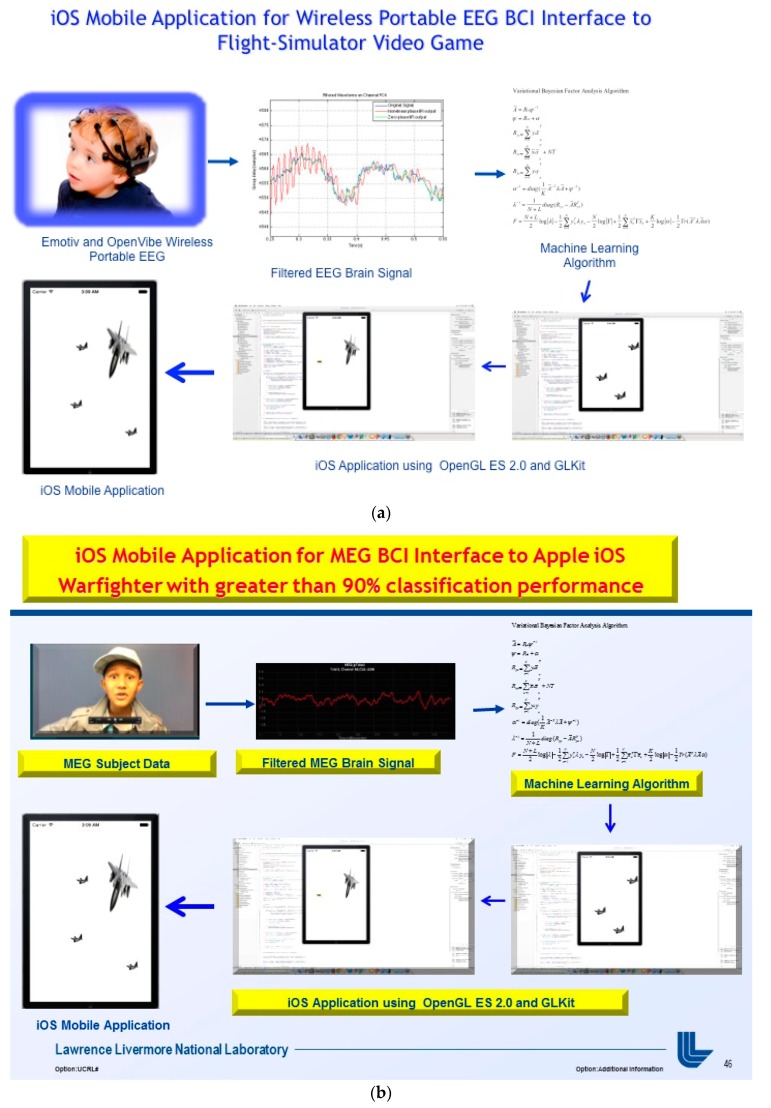

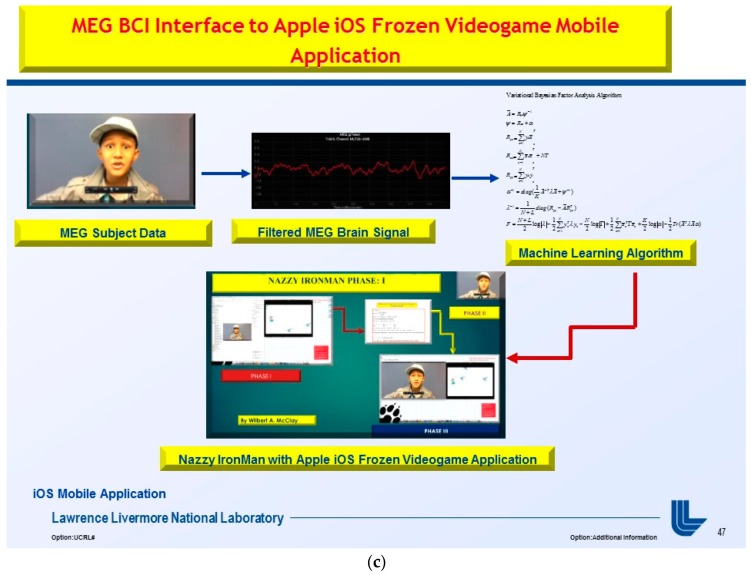
(**a**) iOS Mobile Application of Warfighter Videogame using OpenGL ES 2.0 and GLKit to evade or chase aerial targets. (**b**) iOS Mobile Application of Warfighter Videogame using OpenGL ES 2.0 and GLKit to evade or chase aerial targets can be interfaced to MEG Subject Brain Signal Data with over 90% classification performance. (**c**) Nazzy IronMan with Apple iOS Frozen Videogram Application can be interfaced to with MEG Subject Brain Signal Data with over 90% classification performance.

The Wireless EEG project describes the measurements done with a wireless EEG neuro-helmet while a user is involved in a warfighter simulation while using brainwaves that translate the user’s intentions into actions controlling the warfighter simulator or manually using the iOS UITapGestureRecognizer Class in the OpenGL ES 2.0 and GLKit environment to fire projectiles or control movement which can be done by button-press or from acquired offline Emotiv and OpenVibe EEG brain-wave files while using the following mongodb export function, for example “mongoexport --db brainwaveusers --collection brainsignals --csv --fieldFile fields.txt --out /opt/backups/contacts.csv”, as shown in Figure 29, Figure 30 and Figure 31a below.

We developed a driver that is able to substitute the actions of the BCI as mouse button presses for real-time or non-real-time use in visual simulations. The process was added into warfighter simulator visualization demonstration. By thinking left or right, the user experiences the aircraft turning in the chosen direction. The driver components of the BCI can be compiled into any software and substitute a user’s intent for specific keyboard strikes or mouse button presses to evade or chase aerial targets, as shown in Figure 29 referenced above and Figure 30 below.

The innovation of the Wireless EEG BCI builds upon the BCI technology developed at LLNL, for user intent, which induces certain actions and has myriads of applications. The Wireless EEG BCI allows for people to use their brainwaves and psychological state as control possibilities. In Figure 30 we illustrate this below using the BCI with moving aerial targets in the flow diagram. However, the VBFA machine algorithm performance on EEG data classification is drastically below performance (e.g. approximately less than 60% EEG Subject performance) when compared to greater than 90% performance from MEG Subject data, as illustrated in Figure 31b. This is primarily due to better signal acquisition from the UCSF CTF MEG Scanner with 275 sensor array utilizing Superconducting Quantum Interference Device (SQUIDs) technology in a magnetically shielded room during subject brain signal acquisition and testing [1,22]. In addition, the monetary value of MEG sensors are orders of magnitude greater than EEG, which complements a lower signal-to-noise ratio and lucid signal acquisition for MEG Subject classification. We illustrate the MEG Subject Brainwave classification and implementation on iOS Mobile applications in the subsequent section(s) of this manuscript.

### 3.6. MEG Subject Data BCI iOS Mobile Applications Integration

The MEG Subject Brainwave data demonstrated performance greater than 92% for mid and post- movement thought activity. Thus, the interfacing of iOS Mobile Applications using classified brainwaves as control signals for a user’s intent was a simple implementation, as in Phase I. The interfacing to iOS Mobile Applications was the quintessential usage of the BCI technology since the iOS applications developed in this project were written in Objective-C and the BCI technology in Phase 1 retrieving and utilizing machine learning algorithms [23,24,25] to classify brainwaves was written in C/C++, demonstrated in Figure 31b,c, below. The other aspects of Phase I & Phase II of integrating the MEG Subject’s brainwave performance and videogame analytics into the Hadoop Ecosystem, MongoDB, and Cassandra were written in the Java programming language.

**Figure 32:**
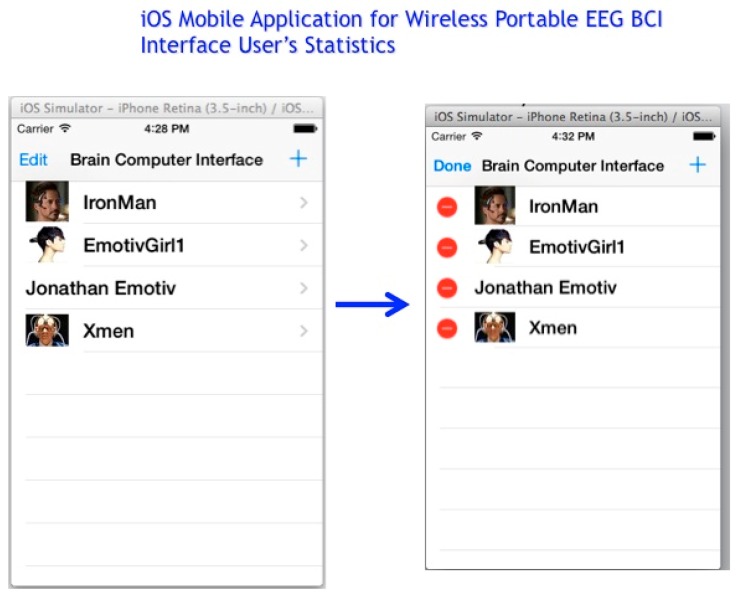
iOS Mobile Application of Warfighter Videogame using OpenGL ES 2.0 and GLKit for online user’s game analytics and dynamic biometrics.

The final and near future phase of the iOS Mobile Application involves the collection of user statistics displayed on an iOS mobile application with output yielded for each user′s videogame analytics’ and dynamic biometric features, such as a user′s UITapGestureRecognizer tap speed or thought movement processes, an illustration of this premise is displayed in Figure 32, below, can be stored and analyzed in a MongoDB database with multiple MEG Subject′s collection or a Cassandra MEG Subject keyspace.

## 4. Conclusions

We are currently extending in several directions the machine-learning module that infers user intent from data. We delineate two of these directions here.

First, the classification algorithm that is described in this paper is based on modeling the data as i.i.d. Gaussian (conditioned on the mixing matrix). However, real MEG data are non-Gaussian and exhibit strong temporal correlations. A model that accounts for those features would describe the data more accurately and could therefore lead to improved classification and performance. We are exploring several extensions of our model, including formulating a time-frequency version to handle temporal correlation and replacing the factor model with a mixture of Gaussian distribution to handle non-Gaussianity.

Second, the present algorithm is designed for binary classification tasks. However, in the majority of BCI applications, the user has several separate and distinct, specific intents [26]. For example, in a flight simulator application, in addition to moving the plane left and right, the user may wish to move it up and down, to rotate it at different angles, and to fly it at different speeds. We are therefore extending our model to handle tasks involving more than two classes.

In Phase II of the BCI project, a non-real-time MEG brainwave CTF files and EEG Emotiv & OpenVibe brainwave files of thought movements [27,28] were analyzed while using signal processing and machine learning algorithms [26] for feature extraction and classification for an iOS mobile application using MongoDB and Cassandra as the MEG/EEG brain signal processing data warehouse. At specific places along the warfighter simulation, the user’s intention to control the warfighter via button presses or hard-wired brainwaves becomes more challenging to avoid aerial targets and fire projectiles. 

**Figure 33:**
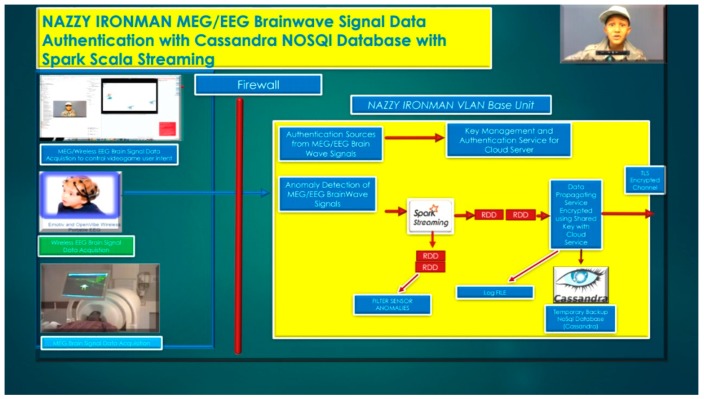
Nazzy Ironman MEG/EEG (Virtual LAN) VLAN Base Unit for Security Authentication.

In the near future, we are developing better machine learning classifier and signal processing algorithms to ameliorate the signal-to-noise ratio, anomaly detection of MEG/EEG brainwave signals, and to perform brain-wave security [29], authentication classification in real-time online internet applications (e.g. NAZZY IronMan MEG/EEG VLAN Base Unit), as illustrated in Figure 33 below; with future outcomes investigating the usage of behavioral monitoring of cognitive state for “thought-actions”, based on historical analysis of brainwave signature data imposed by a given stimulus. 

**Figure 34:**
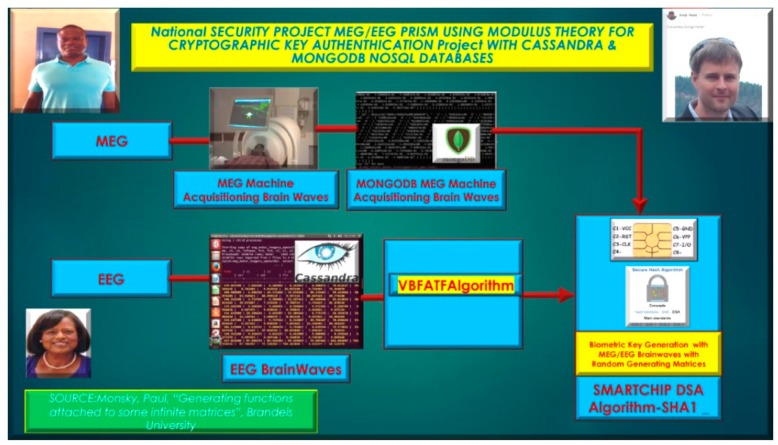
MEG/EEG Cryptographic Key Authentication utilizing MEG/EEG brainwaves with Cassandra and MongoDB NoSQL databases.

Secondly future directions, involve MEG/EEG brainwave signals for cryptographic key authentication using modulus theory based on generating functions utilizing NoSQL databases, such as Cassandra, MongoDB, and components of the Hadoop Ecosystem, such as Hive and Spark Directed Acyclic Graph (DAG) [30,31,32,33]. The usage of Spark DAG and the Spark Machine Learning Library and GraphX to develop biometric key generation based on the utilization of MEG/EEG brain waves for subject authentication. The premise of biometric key generation based on the subject MEG/EEG brainwaves [34] for authentication is based on abstract algebra and combinatoric techniques developed by, Monsky, Paul, “Generating Functions attached to some infinite matrices”, at Brandeis University published in The Electronic Journal of Combinatorics, as illustrated in Figure 34, below.

Our long-term research goal is to develop an MEG/EEG VLAN [35,36,37], Base Unit for Security Authentication of MEG/EEG neuromagnetic brainwave signals [38] to detect and classify authenticated MEG/EEG brainwaves and to reject anomalies and outliers [39] that are demonstrated in Figure 33 [40,41,42,43]. Furthermore, the usage of cryptographic key generation [44,45], based on the biometric authentication of a MEG/EEG subject’s brainwaves will be researched and developed and coupled with DSA SHA-1 algorithm encryption techniques for Cassandra and MongoDB cryptographic biometric databases, as illustrated in Figure 34, above.
